# Data on fungal abundance and diversity in copper and cobalt contaminated tailing soils in Kitwe, Zambia

**DOI:** 10.1016/j.dib.2023.109951

**Published:** 2023-12-12

**Authors:** Leonce Dusengemungu, Cousins Gwanama, Benjamin Mubemba

**Affiliations:** a School of Mathematics and Natural Sciences, The Copperbelt University, Kitwe, Zambia,; b The Copperbelt University, Africa Centre of Excellence for Sustainable Mining, Kitwe, Zambia; c School of Natural Resources, The Copperbelt University, Kitwe, Zambia

**Keywords:** Fungal diversity, ITSF1, Metagenomics, Kitwe

## Abstract

Mining activities in the Zambian Copperbelt Province have led to the release of heavy metal-containing waste, causing contamination in nearby areas. Despite this environmental challenge, limited knowledge exists regarding the mycobiota in copper mine sites. This study investigates fungal community structure in copper(Cu) and cobalt (Co) contaminated soils around decommisioned dams in Kitwe. Metagenomic analysis of the ITSF1 gene amplicons was used for the purpose. The composition of soil fungal communities was characterized, and the findings revealed significant insights. At the phylum level, *Basidiomycota* dominated the fungal profiles in the tailings (64.59%), followed by *Ascomycota* (21.30%), *Glomeromycota* (4.53%), and *Rozellomycota* (0.0275%). Several fungal genera, including *Vanrija*, P*araconiothyrium, Toxicladosporium, Neocosmospora, Septoglomus*, and *Fusarium*, were more abundant in contaminated tailings soils, suggesting their potential in leaching, absorbing, and transforming heavy metals. In contrast, the reference soil at Mwekera National Forest exhibited different dominance patterns with four fungal phyla identified, with *Basidiomycota* and *Ascomycota* dominating most samples. *Glomeromycota*, known for forming arbuscular mycorrhizae with plants, were found in contaminated soils, while *Rozellomycota*, which can serve ecological roles in various ecosystems, were also present. Notable fungal species such as *Aspergillus, Penicillium, Fusarium*, and *Oidiodendron* demonstrated resilience to Cu and Co, the primary contaminants in the Copperbelt.

Specifications Table

**Table:** 

Subject	Metagenomics
Specific subject area	Metagenomics
Type of data	Raw, Raw data FASTQ file
How data were acquired	Soil survey, XRF data (from 0.01 to 15 keV,80 uA) were acquired with the portable device Thermo Scientific Niton XL3t XRF analyzer (ThermoFisher Scientific, Tewksbury, MA, United States), DNA extraction, NGS sequencing.
Data format	Raw data FASTQ file
Experimental factors	ITS region, along with partial gene sequence of 18S rRNA, was amplified using fungal specific primers (ITS1-F) from isolated metagenome
Parameters for data collection	Soil samples were taken on the 16^th^ and 17^th^ August 2022 between 8 am and 1 pm.
Description of data collection	For metagenomic, analysis of ITS1 fungal sequences, a total of ten soil samples were collected from various locations within the selected tailings dams. Two composite soil samples were gathered from the Mwekera Forest to establish controls for comparison. Each soil sample, collected from the uppermost layer (0–25 cm) using a trowel, weighed approximately 500 g and was placed in sealed polyethylene bags. The sampling locations and their respective coordinates are detailed in Table 1, offering precise information about the sources of these critical soil samples.
Data source location	Kitwe
Data accessibility	Repository name: NCBI Data identification number: Accession Number: PRJNA1013767 Direct URL to data: https://www.ncbi.nlm.nih.gov/sra/PRJNA1013767

## 1 Value of the Data

This information offers a thorough analysis and quantitative depiction of the diversity of fungi in Kitwe Tailing dam (TD25), Uchi Tailing dam (TD26) and control soils in Mwekera Natural Forest.

• Based on ITS sequences, data is useful for the comparative analysis of various heavy metal content in tailings and their impact on the overall fungal profile.
• Probabilities of finding novel and uncultured fungi in the tailing dam's metagenome.
• Data provides information on the diversity, distribution, and coexistence of fungi.
• Researchers can use new tools to undertake secondary analysis owing to the availability of raw sequencing data

## 2 Materials and Methods

### 2.1 Study area description, soil collection, and list of plant species

Soil samples (*N* = 10) were collected at three sites within the selected tailings dams (Table 1). Two composite samples at Mwekera Forest were also collected to serve as controls. Approximately 500 g of soil samples were collected from the top layer (0–25 cm) at each location using a trowel, placed in a polyethylene bag, and sealed.

**Table 1:** Sampling location and coordinates.

Sampling Location	Sample code	Coordinates (Latitude & Longitude)
Mwekera Forest Bare Surface area	MW-B1	Lat. -12.86776° Long 28.26223°
Mwekera Forest under (Topsoil of a bare surface area)	MW-B2	Lat. -12.801235° Long 28.218466°
Mwekera Forest (Topsoil of a Planted surface)	MW-P1	Lat-12.849218° Long 28.358872°
Mwekera Forest (Topsoil of a Planted surface)	MW-P2	Lat -12.849218° Long 28.358872°
TD25 Bare soil. (Copper tailings)	TD25-B1,	Lat-12.819904° Long 28.219675°
TD25 Bare soil. (Copper tailings)	TD25-B2	Lat -12.803243° Long 28.21995°
TD25 Top Soil under the thriving trees in the tailings	TD25-P1,	Lat-12.819079° Long 28.223606°
TD25 Top Soil under the thriving trees in the tailings	TD25-P2	Lat-12.821024° Long 28.225504°
TD26 Bare soil. (Copper tailings)	TD26-B1,	Lat-12.82868° Long 28.23249°
TD26 Bare soil. (Copper tailings)	TD26-B2	Lat-12.825224° Long 28.228176°
TD26 Top Soil obtained from the plant areas	TD26-P1	Lat-12.82517° Long 28.228134°
TD26 Top Soil obtained from the planted area	TD26-P2	Lat-12.828595° Long 28.232476°

### 2.2 XRF data acquisition

A portable energy dispersive X-ray fluorescence spectrometer, Thermo Scientific Niton XL3t XRF analyzer (ThermoFisher Scientific, Tewksbury, MA, United States) equipped with a 4 W Rh X-ray tube and a Peltier-cooled Silicon Drift Detector (with 2048 channels, gain of ∼20 eV/channel) was used for XRF data acquisition. The following instrumental conditions were used: (i) X-ray tube voltage of 35 kV and current of 7 μA; (ii) dwell time of 30 s; (iii) no filter was used; and (iv) scans were performed under atmospheric pressure. Three measurements were taken from each soil sample at three different spots, and these were then averaged.

### 2.3 DNA extraction and sequencing

Briefly, genomic DNA was extracted from soil using a ZymoBIOMICS DNA Miniprep kit (Catalog number (Cat #) D4300, Zymo Research) ;(https://www.zymoresearch.com/collections/zymobiomics-dna-kits/products/zymobiomics-dna-miniprep-kit). Extracted gDNA was PCR amplified using a universal primer pair ITS1F for fungal characterization [1]. Resulting amplicons were barcoded with Pacbio M13 barcodes for multiplexing through limited cycle PCR. The resulting barcoded amplicons were quantified and pooled equimolar and AMPure PB bead-based purification step was performed. The PacBio SMRTbell library was prepared from the pooled amplicons following manufacturer's protocol (attached). Sequencing primer annealing and Polymerase binding were done following SMRTlink Link software protocol (online) to prepare the library for sequencing on PacBio Sequel IIe system. The PacBio data analysis was done using DADA2 (https://benjjneb.github.io/dada2/index.html) and qiime2 (https://docs.qiime2.org/2021.11/).

## 3 Data Description

### 3.1 XRF data

**Fig. 1:**
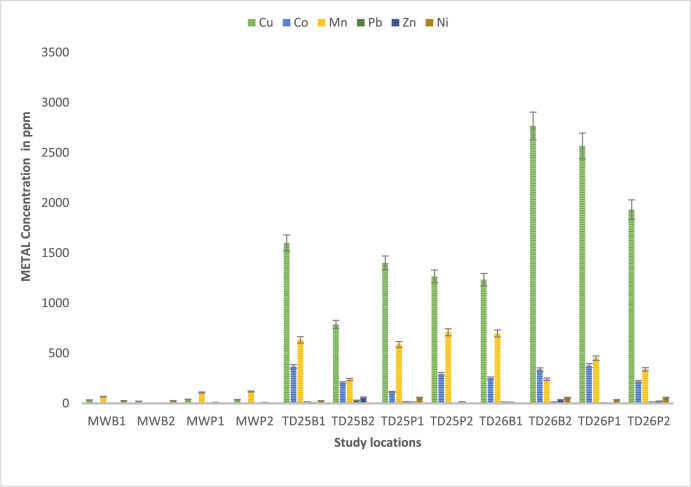
The level of heavy metal concentrations in soil samples of the four sampling sites, namely: Mwekera MW(B1,B2,P1,P2), TD25 (B1,B2,P1,P2), TD26 (B1,B2,P1,P2), and TD10(B1,P1) (mean ± SE).

**Fig. 2:**
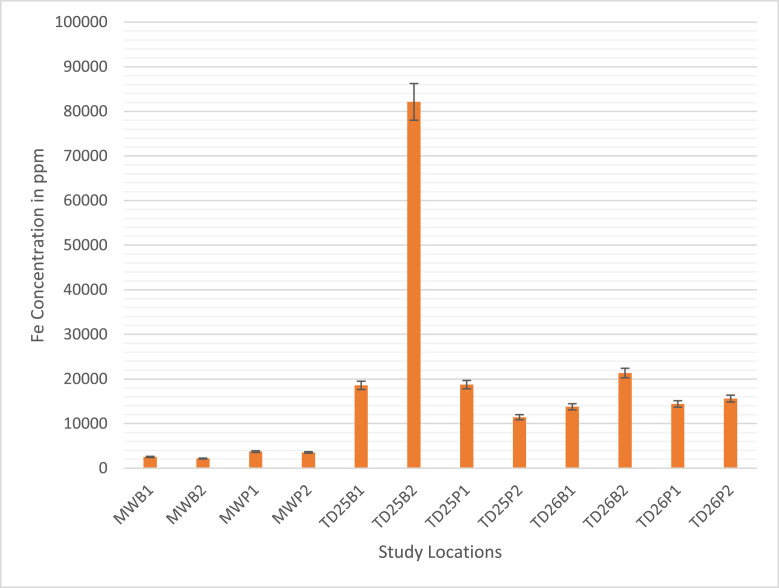
The iron (Fe) concentrations in soil samples of the four sampling sites, namely Mwekera (MWB1, B2, P1, P2), TD25 (B1, B2, P1, P2), TD26 (B1, B2, P1, P2), and TD10 (B1, P1). (Mean ± SE).

In this study, soil samples were collected from Mwekera Natural Forest, and from tailing dams TD25 and TD26. They were then scanned using X-ray fluorescence spectroscopy (XRF). Fig. 1, Fig. 2 shows the heavy metal concentration in composite soils at Mwekera (Bare and Planted Soil), TD25 (Bare and Planted Soil), TD26 (Bare and Planted soil). The concentration is also reported in Supplementary Table 1.

Figs. 1 and 2 show the heavy metal concentration data for Mwekera (MW), TD25 (Kitwe Tailing Dam), and TD26 (Uchi Tailing Dam), revealing varying levels of copper (Cu), cobalt (Co), manganese (Mn), iron (Fe), zinc (Zn), lead (Pb), nickel (Ni), and arsenic (As) across different sampling locations. At TD26 at Uchi Tailing Dam, elevated levels of certain metals such as Cu, Co, and Fe are observed, with distinct variations between sampling points TD26 (B1,B2,P1,P2). The concentrations of heavy metals in TD25, Kitwe Tailing Dam, also exhibit variability across different locations TD25 (B1, B2, P1, P2), with high concentrations of Cu, Fe, and Zn. Mwekera Forest (MW) and TD10 show unique metal composition at different sampling points MW(B1, B2, P1, P2) and TD10 (B1, P1). The average concentrations provide insights into the overall metal content, highlighting potential environmental implications and the need for further investigation into the factors influencing heavy metal distribution in these areas.

### 3.2 A brief overview of plant species diversity at mwekera natural forest and tailing dam areas in the Copperbelt Province of Zambia

Table 2 provides a comprehensive list of plant species commonly observed in the ecosystems of Mwekera Natural Forest and those in the tailing dam areas TD25 and TD26 within the Copperbelt Province of Zambia. This resourceful table offers valuable insights into the botanical composition of these diverse habitats, aiding in the understanding of the ecological dynamics and biodiversity of the studied sites [3,4].

**Table 2:** List of plant species commonly found at Mwekera Natural Forest, TD25 and TD26 on the Copperbelt of Zambia. Noteworthy species in Mwekera Natural Forest (MW) include *Adenia lobata* and *Strychnos spinose*. In TD25, a diverse range of species, such as *Brachystegia boehmii* and *Albizia antunesiana*, is noted, along with the presence of accumulator species like *Syzygium guineense*. TD26 shares species with TD25, including *Albizia antunesiana* and *Syzygium guineense*, while also featuring unique occurrences like *Rhus longipes* and *Peltohorum africanum*, which contribute to enhancing biodiversity. TD10 (Mufulira Tailing Dam) displays a variety of species, including *Albizia antunesiana* and *Rhus longipes*, with additional contributions from species like *Terminalia molii* and *Combretum molle*, enriching the overall plant composition. Key ecological insights highlight notable hyperaccumulators, specifically *Rhus longipes* and *Terminalia molii*, and the presence of accumulators across various species. The role of hyperaccumulators and accumulators is crucial for understanding the ecological functions of these plant species, particularly within the unique context of tailing dam environments [2,5].

Plant Species	MW	TD25	TD26	TD10
Adenia lobata**	x			
*Adenia gummifera (Harv.)* **	x			
Asparagus africanus**	x			
Commelina sp**	x			
Ipomoea sp**	x			
*Trochomeria macrocarpa* **	x			
Strychnos spinosa**	x			
*Thunbergia sp.* **	x			
Cyanotis**	x			
*Tephrosia sp.* **	x			
Brachystegia boehmii ^(^*^)(^**^)^	x			x
Cyphostemma**	x			
Julbernardia paniculata**	x			
Parinari curatelifolia**	x			
*Fadogia cienkowski Schweinf.* **	x			
*Albizia adianthifolia (Schumach.)* **	x			
Brachystegia spiciformis**	x			
*Smilax anceps Willd.* **	x			
Anisophyllea boehmii**	x			
Baphia bequaertii**	x			
*Wahlenbergia sp.* **	x			
Pericopsis angolensis**	x			
Albizia antunesiana^(^*^)(^**^)^	x	x	x	x
Albizia amara*	x			
Syzygium guineense^(^*^)(^**^)^	x	x	x	x
Garcinia livingstonei**	x			
Rhus longipes^(^*^)(^**^)^		x	x	x
Peltohorum africanum*		x	x	x
Lannea discolour*		x	x	x
Senegal polyacantha*		x	x	x
Terminalia molii^(^*^)(^**^)^		x	x	x
Combretum molle*		x	x	x
Mangifera indica*		x	x	
Bauhinia petersiana*		x	x	x
Piliostigma thonningii*		x	x	x
Acacia erioloba*		x	x	x
Curatella Americana*		x	x	x
Cyperus rotundus*		x	x	x
*Persicaria sp.* *				x
*Setaria sp.*				x
*Loudetia sp*				x
*Cymbopogon densiflorus*				x
*Hyparrhenia sp.*				x
*Bulbostylis sp.*				x
*Celosia sp*				x
*Pseudognaphalium luteo-album*				x

**MW** (Mwekera Natura Forest), **TD25** (Kitwe Tailing Dam), **TD26** (Uchi Tailing Dam), **TD10** (Mufulira Tailing Dam), TD4. Hyperaccumulator (**), Accumulators (*)

### 3.3 Fungal diversity at TD25, TD26 and Mwekera forest

The metagenomic data related to the study on ITS1 fungal sequences in soil samples collected from various locations, including controls from Mwekera Forest is available on the NCBI repository. The raw data, in FASTQ file format, can be accessed with the identification number PRJNA1013767 at the following URL:https://www.ncbi.nlm.nih.gov/sra/PRJNA1013767](https://www.ncbi.nlm.nih.gov/sra/PRJNA1013767).

**Fig. 3:**
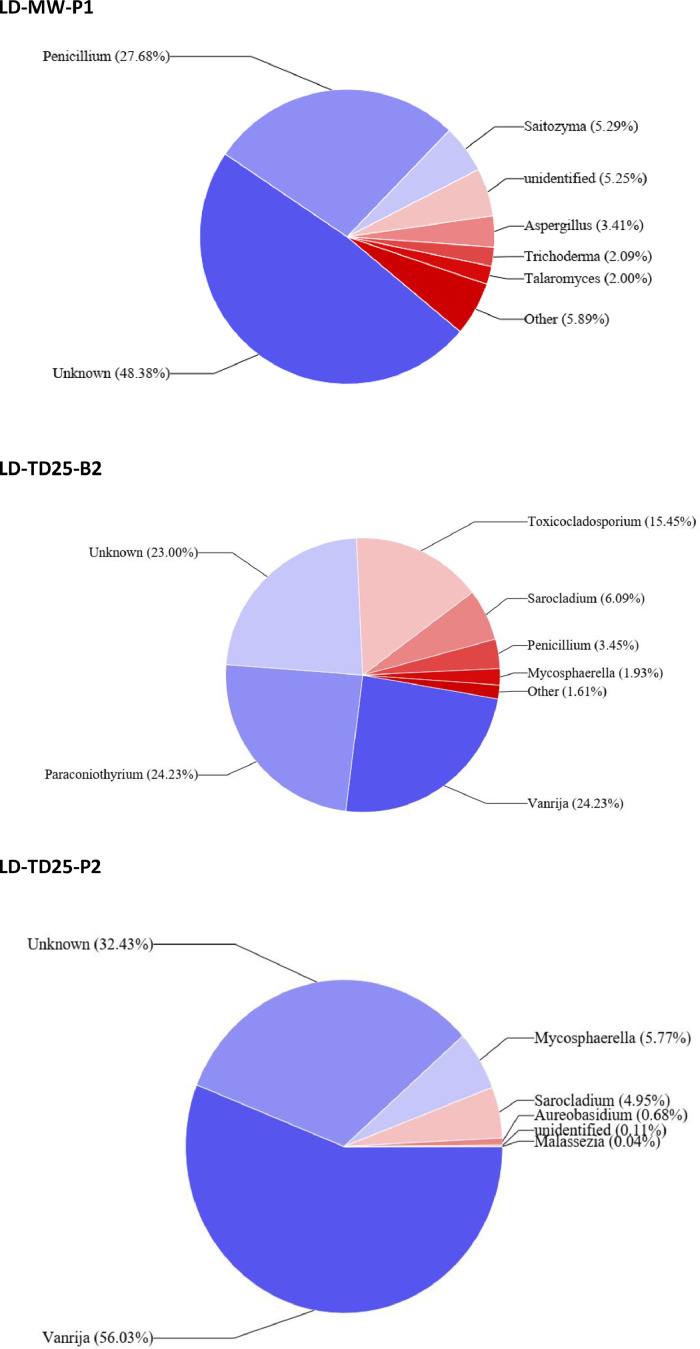

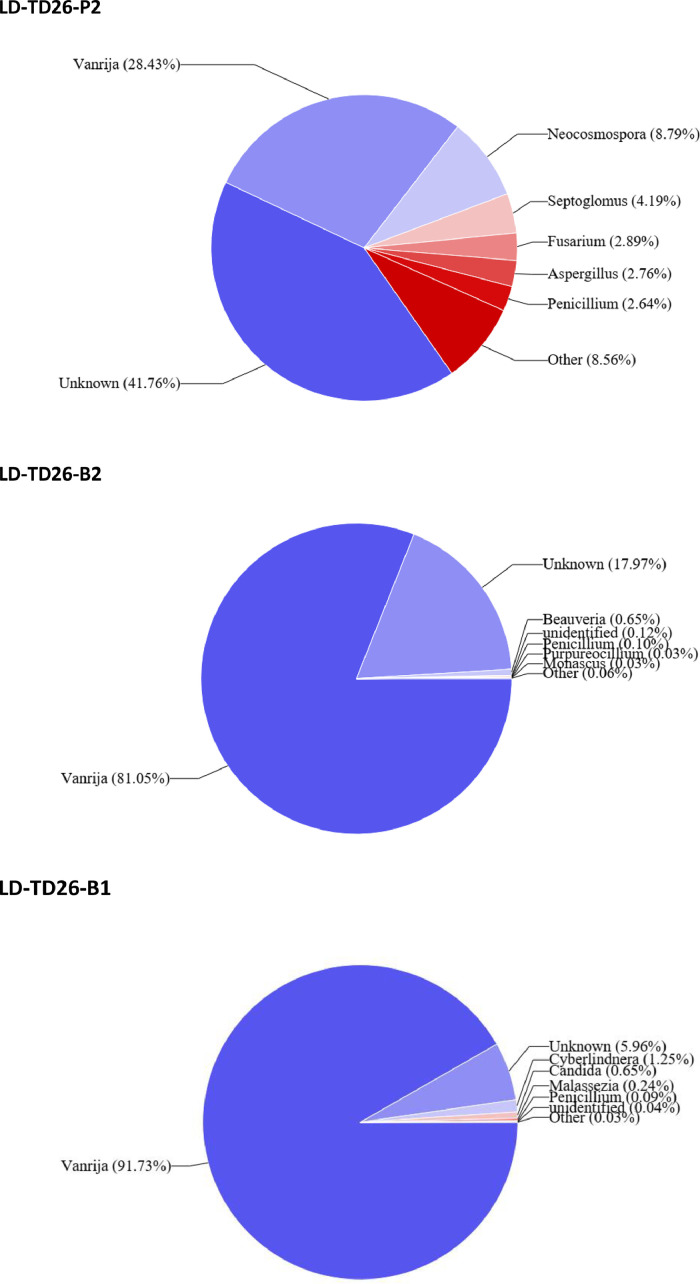
Fungal communities associated with contaminated surface soils are major alternatives for bioremediation initiatives. The data presented in this figure are at the genus level, providing a detailed insight into the specific fungal genera contributing to the remediation potential of the contaminated soil environments. Using fungal ITSF1 community profiles, we may compare the more abundant taxa in polluted (TD25, TD26) vs. uncontaminated surface samples from Mwekera Forest. **MW** (Mwekera Natura Forest) (P1: Soil from planted area), **TD25** (Kitwe Tailing Dam) (B2: Soil from Bare surface soil, P2: Soil from planted surface soil), **TD26** (Uchi Tailing Dam): B2, B1: Soil from bare surface area), P2: Soil from planted surface soil.

Four fungal phyla were identified in this study from all soil samples tested. Among them, *Basidiomycota* and *Ascomycota* dominated. However, *Basidiomycota* predominated in metal-contaminated soils with the exception of one soil sample from TD25 where *Ascomycota* predominated. *Ascomycota* was the most prevalent in reference soil (LD-MW) (Fig. 3).

At family level the average 16S rRNA soil fungal profiles in the tailings were dominated by *Basidiomycota* (64.59%), *Ascomycota* (21.302%), *Glomeromycota* (4.53%), and *Rozellomycota* (0.0275%) (Fig. 3, LD-TD25-B2, LD-TD25-P2, LD-TD26-P2, B1, B2). At the genus level, the 16S rRNA soil fungal profiles at Mwekera National Forest (LD-MW-P1) were consisted of *Penicillium* (27.68%), *Saitozyma* (5.29%), *Aspergillus* (3.41%), *Trichoderma* (2.09%), *Talaromyces* (2%), and the unidentified and unknown counts for 53.63%. *Vanrija* (24.23%) *Paraconiothyrium* (24,23%) dominated the genus fungal profiles of bare soil at LD-TD25-bz, followed by*Mycosphaerella* (1.93%), *Penicillium* (3.45%), Sarocladium (6.09%), *Toxicocladosporium*(15.45%) and unknown (23%). In comparison, the fungal profiles of the topsoil from the planted area at TD25-P2 were dominated by *Vanrija* (56.03%), unknown and unidentified (32.54%), *Mycosphaerella* (5.77%), *Sarocladium* (4.95%), *Aureobasidium* (0.68%), *Malassezia* (0.04%). *Vanrija* (28.43%), *Neocosmospora* (8.79%), *Septoglomus* (4.19%), *Fusarium* (2.89%), *Aspergillus* (2.76%), *Penicillium* (2.64%) and unknown (41.76%) dominated the fungal profiles from the planted top soil of LD-TD26-P2 at the genus level. The fungal profile of the topsoil from the bare area at LD-TD 26-B1 was dominated by *Vanrija* (91,73%), unknown (5.96%), *Cyberlindera* (1.25%), *Candida* (0.65%), *Malassezia* (0.24%), *Penicillium* (0.009%), unidentified (0.04%), *Beauveria* (0.65%), *Purpureocillium* (0.03%), *Monascus* (0.003%). Several fungal genera, namely *Vanrija, Paraconiothyrium, Toxicladosporium, Neocosmospora, Septoglomus*, and *Fusarium*, were more abundant in contaminated soil of the tailings dams (LD-TD 25, LD-TD 26) than in uncontaminated soils of Mwekera National Forest (LD-MW)

## Ethical Approval

It is not required for this study the research did not involve humans or animals.

## CRediT authorship contribution statement

**Leonce Dusengemungu:** Conceptualization, Data curation, Formal analysis, Funding acquisition, Investigation, Methodology, Validation, Visualization, Writing – original draft, Writing – review & editing. **Cousins Gwanama:** Supervision, Writing – original draft, Writing – review & editing. **Benjamin Mubemba:** Supervision, Writing – original draft, Writing – review & editing.

## Data Availability

Fungal communities' ITSF1 gene profiles in soil. Raw sequence reads (Original data) (NCBI)

## Appendix Supplementary materials

Concentrations (ppm) of various elements in tailing dams and their averages Supplementary Table 1
